# Strategies to safely target widely expressed soluble adenylyl cyclase for contraception

**DOI:** 10.3389/fphar.2022.953903

**Published:** 2022-08-25

**Authors:** Jacob Ferreira, Lonny R. Levin, Jochen Buck

**Affiliations:** Department of Pharmacology, Weill Cornell Medicine, New York, NY, United States

**Keywords:** cAMP, sperm motility, capacitation, fertility, adcy10, birth control

## Abstract

In humans, the prototypical second messenger cyclic AMP is produced by 10 adenylyl cyclase isoforms, which are divided into two classes. Nine isoforms are G protein coupled transmembrane adenylyl cyclases (tmACs; ADCY1-9) and the 10th is the bicarbonate regulated soluble adenylyl cyclase (sAC; ADCY10). This review details why sAC is uniquely druggable and outlines ways to target sAC for novel forms of male and female contraception.

## Introduction

Cyclic adenosine monophosphate (cAMP), the prototypical second messenger, is a key player facilitating signal transduction throughout the bacterial and animal kingdoms. In mammalian cells, cAMP-dependent signaling is involved in various biological processes such as development, proliferation, and apoptosis via its various effectors, which include Protein Kinase A (PKA), exchange proteins activated by cAMP (EPACs), and cyclic nucleotide-gated channels (Bos, 2003; Kopperud et al., 2003; Kamenetsky et al., 2006; Biel, 2009). With the recognition that this second messenger has many divergent roles, oftentimes within a single cell type, it was appreciated that cAMP is compartmentalized into discreet signaling microdomains. Microdomains are defined by a specific adenylyl cyclase (AC) to produce cAMP and a phosphodiesterase (PDE) to degrade cAMP to prevent the signal from impacting neighboring microdomains and to limit its duration (Scott et al., 2013; Zaccolo et al., 2021). Thus, if one can selectively target the relevant microdomain, the cAMP cascade provides a plethora of targets for treating diseases, including receptors regulating AC activity, the ACs themselves, PDEs, and cAMP effector proteins. For example, increasing cAMP signaling in a microdomain can be pharmacologically manipulated by activating the appropriate AC or the receptor regulating it, or by inhibiting the relevant PDE. Similarly, decreasing cAMP signaling can be accomplished by inhibiting the AC or its receptor, activating the PDE, or blocking the action of the specific cAMP effector. Many therapeutics work via cAMP by modulating the hormone and neurotransmitter receptors regulating AC activity (Sriram and Insel, 2018), and recently, a number of therapeutics directly elevate cAMP within specific microdomains via isoform-selective PDE inhibitors (Baillie et al., 2019). In contrast, there are no approved therapeutics targeting individual AC isoforms.

**FIGURE 1:**
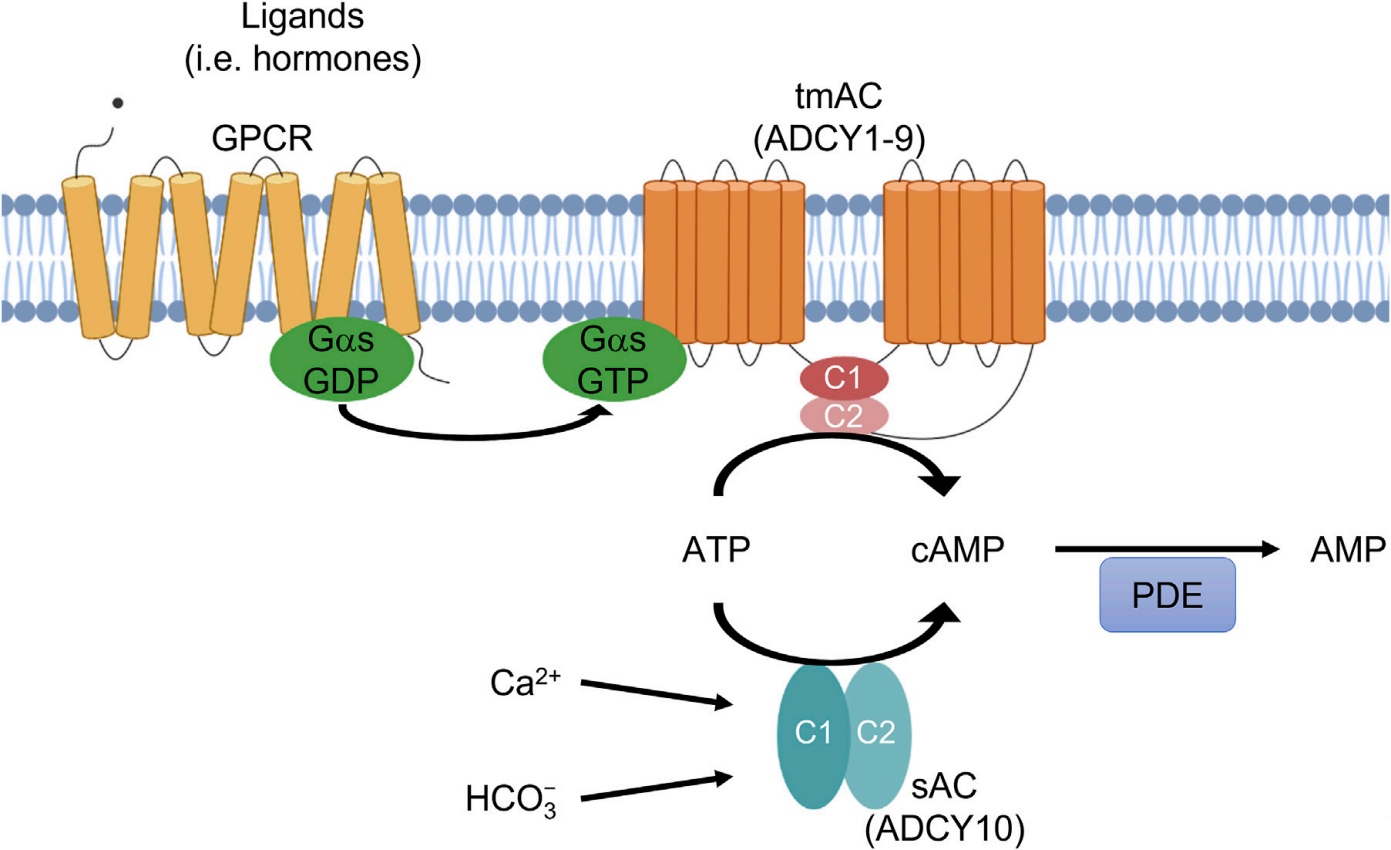
Two sources of cAMP in mammalian cells. ADCY1-9 are transmembrane adenylyl cyclases (tmACs) regulated by G-proteins. ADCY10 is soluble adenylyl cyclase (sAC) regulated by HCO_3_^−^ and Ca^2+^ ions. Phosphodiesterases (PDEs) degrade cAMP. sAC and tmACs both produce cAMP which has distinct roles.

In humans, there are 10 genes encoding AC isoforms, ADCY1-10, which can be divided into two classes (Figure 1): nine genes (ADCY1-9) encode transmembrane adenylyl cyclases (tmACs) while the most recently identified gene (ADCY10) encodes the soluble adenylyl cyclase (sAC) (Buck et al., 1999). The transmembrane adenylyl cyclases are regulated by heterotrimeric G proteins and are responsible for cAMP signaling downstream from hormones and neurotransmitters modulating G protein coupled receptors (GPCRs). In contrast, sAC has no predicted transmembrane domains, is not regulated by heterotrimeric G proteins, and is localized to different parts of the cell including the cytosol, mitochondria and nucleus (Zippin et al., 2003). Originally, biochemical studies identified a soluble AC activity, which was thought to be restricted to male germ cells in the testis (Braun and Dods, 1975; Neer, 1978). Its activity first appeared concurrently with the development of spermatids in rats (Braun and Dods, 1975; Braun et al., 1977) and humans (Gordeladze et al., 1982) and was present in testis fractions enriched for spermatids (Braun et al., 1977; Gordeladze et al., 1981). sAC activity is directly regulated by HCO_3_^−^ and Ca^2+^ ions (Chen et al., 2000; Jaiswal and Conti, 2003; Litvin et al., 2003; Kleinboelting et al., 2014), and its activity is sensitive to physiologically relevant fluctuations in its substrate, ATP (Zippin et al., 2013). Because it is molecularly and biochemically distinct from other mammalian nucleotidyl cyclases, sAC defines cAMP signaling cascades in mammalian cells independent from the widely studied, hormone-responsive tmACs (Kamenetsky et al., 2006; Tresguerres et al., 2011; Wiggins et al., 2018). Consistent with its biochemical activity profile, sAC is most abundantly expressed in male germ cells (Buck et al., 1999; Sinclair et al., 2000); however, sAC is also widely expressed at lower levels in somatic tissues (Buck et al., 1999; Geng et al., 2005). sAC-generated-cAMP has many functions that are distinct from tmAC-generated-cAMP (Wiggins et al., 2018; Ostrom et al., 2022); including motility and capacitation of sperm (Esposito et al., 2004; Hess et al., 2005; Akbari et al., 2019; Balbach et al., 2021), and regulation of liver inflammation and fibrosis (Wang et al., 2020), pH homeostasis (Tresguerres et al., 2010), oxidative phosphorylation (Acin-Perez et al., 2009; Di Benedetto et al., 2013; Lefkimmiatis et al., 2013), lysosomal function (Rahman et al., 2016), ciliary beating frequency (Schmid et al., 2007; Chen et al., 2014), glucose homeostasis (Zippin et al., 2013; Holz et al., 2014), and intraocular pressure (Lee et al., 2011; Gandhi et al., 2017).

### Soluble adenylyl cyclase can be selectively targeted by small molecule inhibitors

**FIGURE 2:**
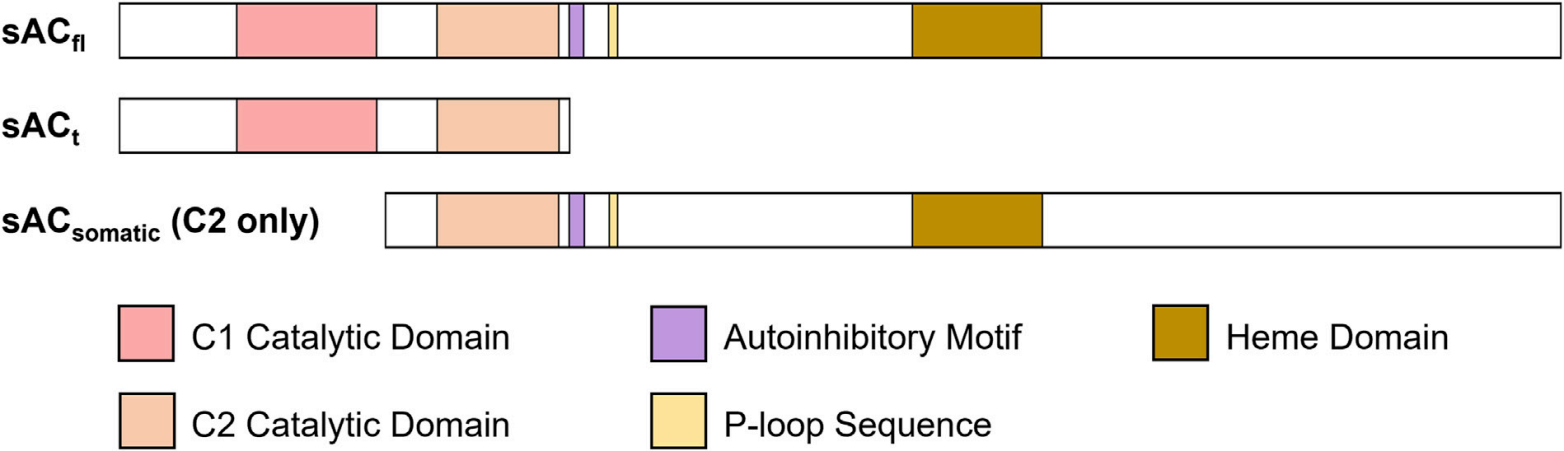
Different isoforms of sAC with catalytic and functional domains highlighted. sACt and sACfl are present in testis and sperm, while sACsomatic appears to be ubiquitously expressed.

In contrast to the tmACs which generate isoform diversity via distinct genes (Ostrom et al., 2022), in humans and rodents, sAC isoform diversity arises from splice variants of the single ADCY10 locus (Figure 2) (Jaiswal and Conti, 2001; Geng et al., 2005; Schmid et al., 2007; Farrell et al., 2008; Middelhaufe et al., 2012; Karczewski et al., 2020). When sAC was first purified, we isolated two independent cDNAs representing two alternatively spliced isoforms of sAC (Buck et al., 1999; Jaiswal and Conti, 2001). These two transcripts correspond to a full-length sAC (sACfl), encoding a 187 kDa protein, and a truncated sAC (sACt), encoding a 48 kDa protein, both of which are expressed in mouse testis (Hess et al., 2005). sACt is predominantly comprised of two homologous catalytic domains (C1 and C2) (Buck et al., 1999; Jaiswal and Conti, 2001), and because it is lacking an autoinhibitory domain present in longer isoforms, it exhibits 10-fold higher specific activity than sACfl (Chaloupka et al., 2006). Subsequent molecular cloning and 5’ Rapid Amplification of cDNA Ends (RACE) experiments identified an alternate start site which generates sAC isoforms missing the first catalytic domain. Because these sAC-C2 only isoforms have not yet been biochemically characterized, all work describing sAC physiological regulators and pharmacological inhibitors refers to the sAC C1-C2 isoforms abundantly expressed in testis and sperm.

After cloning sAC, we appreciated the need for a selective sAC inhibitor to distinguish between sAC- and tmAC-specific physiologic processes. In a high throughput screen (Hess et al., 2005), we identified KH7, a modestly potent (IC_50_ ∼3 µM) sAC-selective inhibitor which has proven instrumental in many cellular studies (Bitterman et al., 2013; Steegborn, 2014; Wiggins et al., 2018). However, KH7 is not a very drug-like molecule and has off-target effects resulting in sAC-independent cytotoxicity (Tian et al., 2011; Di Benedetto et al., 2013). To identify a more suitable candidate for therapeutic development, we performed a new high throughput screen and identified LRE1, a non-toxic sAC inhibitor with a novel chemical structure (IC_50_ ∼3 µM) (Ramos-Espiritu et al., 2016). Crystal structures of sAC/LRE1 complexes revealed that the 2-amino-6-chloropyrimidine moiety of the compound occupies the bicarbonate binding site (BBS) (Ramos-Espiritu et al., 2016). Consistently, LRE1 inhibition is competitive with bicarbonate but non-competitive with substrate ATP, rendering the compound the first truly allosteric BBS-targeting sAC inhibitor. In tmACs, the pseudo-symmetrical regulatory binding site analogous to the sAC BBS binds forskolin, a general activator of tmACs (Hurley, 1999). Forskolin is inert towards sAC (Buck et al., 1999) because it is too bulky to fit in the sAC BBS (Kleinboelting et al., 2014). Conversely, the forskolin binding site of individual tmAC isoforms appears to be able to accommodate LRE1, which is a weak activator of certain tmACs (Ramos-Espiritu et al., 2016). We took advantage of the drug design expertise of a unique public-private partnership (Meinke, 2022) to improve the potency, selectivity, and drug-like characteristics of LRE1. Ultimately, these efforts increased potency for sAC over 10,000-fold with corresponding increased efficacy in cell-based assays, diminished cross-reactivity with other mammalian nucleotidyl cyclases, and no significant cytotoxicity (Fushimi et al., 2021).

### Soluble adenylyl cyclase can be safely targeted for contraception

A subset of the physiological functions ascribed to sAC have therapeutic implications (Wiggins et al., 2018). Chief among these is sAC’s role in male fertility (Esposito et al., 2004; Hess et al., 2005; Akbari et al., 2019; Balbach et al., 2021). sAC, specifically isoforms containing both C1 and C2 domains (Hess et al., 2005), is most abundant in testis and sperm and is essential for sperm to fertilize an egg. After being produced in the testis, mammalian sperm are stored in the cauda region of the epididymis where they are morphologically mature but unable to fertilize an egg. The cauda region is unique because it is characterized by low pH (i.e. 6.5–6.8 vs. 7.4 at physiological conditions) and low HCO_3_^−^ concentration (i.e., 2–7 mM vs. 25 mM). These unique conditions maintain sperm in a dormant state (Levine and Kelly, 1978). Upon ejaculation, sperm encounter semen containing high concentrations of HCO_3_^−^ and Ca^2+^ (Wennemuth et al., 2003; Carlson et al., 2005), which initiate motility and a post-ejaculation maturation process termed capacitation (Austin, 1951; Chang, 1951). The higher levels of HCO_3_^−^ and Ca^2+^ in semen synergistically activate sAC in sperm which rapidly increases cAMP production and initiates sperm capacitation (Visconti et al., 1995; Visconti et al., 2002; Buffone et al., 2014; Balbach et al., 2018). Capacitation-induced changes include activation of motility, specifically, increased beat frequency and altered, asynchronous beating known as hyperactivation. These changes are essential for sperm to pass through the cervix to leave the inhospitable environment of the vagina and enter the permissive environment of the uterus (Visconti et al., 1995; Visconti et al., 2002; Suarez and Pacey, 2006; Buffone et al., 2014; Balbach et al., 2018).

sAC’s essential role in male reproduction has been validated genetically (Esposito et al., 2004; Hess et al., 2005; Akbari et al., 2019; Balbach et al., 2021) and pharmacologically (Hess et al., 2005; Mannowetz et al., 2012; Ramos-Espiritu et al., 2016; Balbach et al., 2021; Ferreira et al., 2021) in both mice and men. Two different strains of sAC knockout (KO) mice show male-specific sterility; their sperm are immotile and unable to fertilize an oocyte *in vitro* (Esposito et al., 2004; Hess et al., 2005; Xie et al., 2006; Chen et al., 2013). sAC is also among the rare instances where a human mutation reveals a desired phenotype for a therapeutic approach. Two middle-aged infertile men were identified as homozygous for a frame shift mutation interrupting the catalytic domains of sAC (Akbari et al., 2019). Sperm from these men were immotile, similar to sperm from sAC KO mice (Esposito et al., 2004; Hess et al., 2005; Xie et al., 2006; Balbach et al., 2021), and their motility defect could be rescued by addition of membrane permeable cAMP (Akbari et al., 2019). sAC’s essential role in fertility, and as a target for contraception, has also been validated pharmacologically in humans and mice. Multiple, structurally independent, sAC-specific inhibitors prevent the capacitation-induced changes in mouse and human sperm, including stimulation of cAMP production, protein kinase A (PKA) activation, alkalinization, increased beat frequency, hyperactivated motility, and ability to undergo a physiologically induced acrosome reaction (Hess et al., 2005; Mannowetz et al., 2012; Ramos-Espiritu et al., 2016; Balbach et al., 2021; Ferreira et al., 2021). sAC-specific inhibitors also prevent *in vitro* fertilization in mice (Ramos-Espiritu et al., 2016; Balbach et al., 2021). These data validate sAC inhibitors as potential contraceptives (Buffone et al., 2014; Balbach et al., 2020; Balbach et al., 2021); however, the question remains whether they can be administered safely and effectively to people.

Historically, the only targets in sperm pursued for contraceptive development were those that were exclusively expressed in testis (Amory, 2016; O'Rand et al., 2016); it had been assumed that expression of a target like sAC in somatic tissues would lead to unsurmountable mechanism-based side effects. However, three advances question this dogma:

1) sAC isoforms expressed in somatic tissues may differ from the C1-C2 containing isoforms abundantly expressed in testis and sperm.
2) Advances in vaginal delivery methods afford the opportunity to supply sAC inhibitors designed for topical use to selectively block sperm functions in the female reproductive tract with little systemic exposure.
3) The appreciation that besides male infertility, the phenotypes of sAC loss in humans and mice are due to chronic loss, which suggests acute acting inhibitors can safely provide “on-demand” contraception in men. Such contraceptives would be taken only as needed to temporarily render men infertile.

#### Targeting sperm specific soluble adenylyl cyclase isoforms

As described above, the biochemically characterized and pharmacologically targeted sAC isoforms, sACt and sACfl are expressed in testis and sperm (Jaiswal and Conti, 2001; Hess et al., 2005). According to a recent human gene expression profiling database (Karczewski et al., 2020), these C1-C2 containing isoforms are not found in somatic tissues; instead somatic tissues seem to use an alternate start site (Jaiswal and Conti, 2001; Geng et al., 2005; Schmid et al., 2007; Farrell et al., 2008; Middelhaufe et al., 2012; Karczewski et al., 2020) to express only C2-domain containing isoforms from the ADCY10 locus (Figure 2). cDNAs encoding sAC-C2 isoforms have been isolated from various organs, including the kidneys, small intestine (Geng et al., 2005) and lungs (Schmid et al., 2007) but more research needs to be done to fully elucidate their activity, expression, distribution, and localization. Like all mammalian nucleotidyl cyclases, sAC isoforms are Class III ACs, and their catalytic center is formed at the interface between two catalytic domains. In C1-C2 containing sACt and sACfl, the active site is formed from the intramolecular dimerization of two structurally similar but unique catalytic domains (i.e., C1 and C2) (Tang and Hurley, 1998; Wiggins et al., 2018). The interface of these domains contains a catalytic site which binds substrate ATP and a pseudo-symmetrical degenerate “active” site that is catalytically inactive and binds the sAC-specific activator HCO_3_^−^ (BBS) (Kleinboelting et al., 2014; Steegborn, 2014). Like C1-C2 isoforms of sAC, tmACs’ active sites are formed via intramolecular dimerization of two related catalytic domains, but guanylyl cyclases (GCs) employ different molecular architectures (Tesmer et al., 1997; Kamenetsky et al., 2006). The various genes encoding GCs all contain only a single catalytic domain; soluble GCs form active sites via intermolecular dimerization of subunits containing structurally similar but unique catalytic domains while membrane GCs form intermolecular homodimers for activity. It remains unclear how sAC-C2 isoforms form an active cyclase. In C1-C2 sAC isoforms, the C1 domain contributes key catalytic residues that would be absent if sAC-C2 isoforms were to homodimerize, and no known C1-like binding partner has yet been identified for sAC-C2. The newest generation of potent and selective inhibitors were generated via structure-based drug design using a crystal structure of human C1-C2 containing sAC (Fushimi et al., 2021). These more potent inhibitors, which fill both the BBS and the active site formed at the interface of C1 and C2, making contacts in both domains, were used to validate sAC as a contraceptive target. Thus, it remains possible that these inhibitors are specific for the sAC isoform expressed predominantly, if not exclusively, in sperm; however, this strategy awaits further information about tissue distribution and characterization of other sAC isoforms, including the sAC-C2 isoforms which are hypothesized to be the isoforms expressed in somatic tissues.

#### Intravaginal drug delivery

Leveraging chemical modifications to tailor routes of administration or pharmacokinetic properties is a standard technique to target specific tissues (e.g., topical drugs which work locally with little systemic exposure) or organs (e.g., using first pass metabolism to concentrate drug in liver (Niemi, 2007; Tu et al., 2013; Zhou et al., 2015; Li et al., 2019) or increasing urinary excretion to target the kidneys). For contraception, because sAC is essential for hyperactivated motility of human sperm (Balbach et al., 2021), delivering a sAC inhibitor to the vagina would block ejaculated sperm from progressing beyond the cervix. Thus, inhibiting sAC in sperm inside the vagina would trap them in this inhospitable environment and prevent fertilization. This idea is already validated *in vitro* with post-ejaculated sperm (Balbach et al., 2021; Ferreira et al., 2021). Specifically, sAC inhibitors interrupt capacitation, inhibit progressive motility, and block acrosome reaction in human sperm when added post-ejaculation (i.e., after capacitation has begun), which is exactly when a vaginally delivered contraceptive would encounter activated sperm. sAC inhibitors suitable for intravaginal delivery would have to be tested for stability and efficacy in the acidic vagina, and they would be designed to include elements providing metabolic instabilities upon reaching systemic circulation. Metabolic instability in the bloodstream would limit distribution of compound absorbed through the vaginal mucosa and prevent inhibitors from affecting other organs. Such, topical sAC inhibitors, delivered *via* intravaginal devices (i.e., rings, films, gels, or suppositories), would provide non-hormonal female contraception with diminished concerns for systemic adverse effects.

Topical administration in the female reproductive tract is an area of active research in the contraception field. Many topical formulations are well tolerated by women and each has their own advantages. Currently, there are multiple FDA-approved, hormone-based contraceptives in the form of vaginal rings (i.e. NuvaRing, Annovera). NuvaRing is a once monthly vaginal ring (FDA Insert, 2001), whereas Annovera is used for a full year (FDA Insert, 2018). Using a sAC inhibitor as the active drug for contraception provides the distinct advantage of being non-hormonal. Similar to existing intravaginal devices, sAC-based contraceptives could be formulated to be slow releasing rings providing long-term coverage for contraception, or as gels, films or suppositories, which would provide contraceptive protection acutely and used on-demand (i.e., only when necessary).

Vaginal delivery of a topical sAC inhibitor also offers an opportunity for developing multi-protection technology (MPTs) where contraception is provided along with protection from sexually transmitted infections (STIs) (Clark et al., 2014). Recent research has focused on developing vaginal topical delivery systems for the treatment of HIV-1 infections and other STIs (Woolfson et al., 2006; Robinson et al., 2018; Nel et al., 2021). A sAC inhibitor-based contraceptive would be ideal for incorporation into an MPT as it is non-hormonal and could be combined with other drugs in the topical formulation for multiple indications. Such a combination could be used long-term or acutely for short-term contraception and prevention of STIs.

#### Acute dosing

As mentioned above, sAC as a contraceptive target is genetically validated in both mice and men. KO mice (Esposito et al., 2004; Hess et al., 2005; Xie et al., 2006; Chen et al., 2013) and humans (Akbari et al., 2019) harboring sAC inactivating mutations are male specific sterile. In addition to being an uncommon example where a human mutation validates a therapeutic approach, the phenotypes of mice and men with sAC deletions reveal a strategy for an innovative paradigm for oral contraception. sAC KO humans and mice live relatively normal lives with modest adverse effects. Other than male-specific sterility, all known phenotypic consequences of sAC loss in mice and humans are “conditional” and dependent upon prolonged absence of sAC (Wiggins et al., 2018; Balbach et al., 2020). sAC KO men have increased propensity to form kidney stones (Akbari et al., 2019), which require years to form. Similarly, sAC KO mice have modestly increased intraocular pressure (Lee et al., 2011), which could eventually cause glaucoma but again, this would require years of sAC absence. These phenotypes indicate that intermittent dosing of an acutely acting sAC inhibitor would not elicit adverse effects, and suggest a paradigm of “on-demand” contraception, where a man would be rendered temporarily infertile minutes after a single dose of a fast-acting, short-lived inhibitor. With an on-demand birth control for men, the contraceptive effect as well as any other potential consequences would be gone after a few hours.

A similar on-demand strategy was successful implemented with erectile dysfunction drugs. Like sAC, the cGMP-specific phosphodiesterase 5 (PDE5) is widely expressed (Bender and Beavo, 2006), yet acute PDE5 inhibitors (i.e., sildenafil, vardenafil) are sufficiently safe and used to treat erectile dysfunction (FDA Insert, 1998). In fact, the PDE5 inhibitor tadalafil lasts longer (∼17 h) (FDA Insert, 2003) and is sufficiently safe for chronic use (Montorsi et al., 2004). Ideally, a sAC inhibitor for contraception would be taken 30–60 min before sexual activity and provide safe and effective contraception for 4–6 h. A sAC based on-demand contraceptive would change the contraception field, it would be a pharmacologic option that is non-hormonal while allowing for complete freedom for the individual to take the drug only when necessary.

On-demand oral contraception by sAC inhibitors may also be possible for females. For males, taking the drug before sexual activity, would inhibit sAC in epididymal sperm to block their ejaculation-induced activation. sAC inhibitors could also be formulated to achieve efficacious concentrations throughout the female reproductive tract. A female would take such a sAC inhibitor orally shortly before, or perhaps soon after, sex to interrupt capacitation and motility of post-ejaculated sperm. sAC inhibition would prevent sperm from progressing through the female reproductive tract to reach and fertilize the egg. Combining both ideas could lead to a “couples’ pill” where both partners take their respective drug at the same time to maximize contraceptive efficacy. A “couples’ pill” would enhance compliance by engaging both partners, and it could increase adoption by making the choice for contraception a joint decision.

## Discussion

There is large unmet need in contraception and new methods must be developed, especially contraceptive methods which are non-hormonal and which provide additional choices for men. Currently, contraception is largely the responsibility of women; of all modern forms of contraception available, all but two are for women. The most widely used options for women are hormone-based pharmacologic methods (pills, patches, injectables, or rings), which have significant drawbacks including adverse effects and compliance issues (La Vecchia et al., 2001; Cushman et al., 2004; Kulier et al., 2004; Huber et al., 2006; Peachman, 2018; Cooper and Mahdy, 2021). Similarly, the only two options for men, surgical vasectomy or condoms, also suffer from compliance and inconvenience issues (Amory, 2016). Here we describe three innovative strategies for inhibiting the bicarbonate-regulated sAC for contraceptive effect to fulfill this need: (1) targeting organ specific sAC isoforms; (2) leveraging chemical modification to alter route of administration and pharmacokinetic properties to have sAC-based drugs exert their effect only on the organ of interest; (3) using acute dosing instead of chronic dosing.

## Data Availability

The original contributions presented in the study are included in the article/supplementary material, further inquiries can be directed to the corresponding author.
